# Effects of Aging and Tocotrienol-Rich Fraction Supplementation on Brain Arginine Metabolism in Rats

**DOI:** 10.1155/2017/6019796

**Published:** 2017-11-19

**Authors:** Musalmah Mazlan, Hamizah Shahirah Hamezah, Nursiati Mohd Taridi, Yu Jing, Ping Liu, Hu Zhang, Wan Zurinah Wan Ngah, Hanafi Ahmad Damanhuri

**Affiliations:** ^1^Faculty of Medicine, Universiti Teknologi MARA, Sungai Buloh Campus, Sungai Buloh, Malaysia; ^2^Department of Biochemistry, Faculty of Medicine, Universiti Kebangsaan Malaysia, Kuala Lumpur, Malaysia; ^3^Department of Anatomy, University of Otago, Dunedin, New Zealand; ^4^School of Pharmacy, University of Otago, Dunedin, New Zealand

## Abstract

Accumulating evidence suggests that altered arginine metabolism is involved in the aging and neurodegenerative processes. This study sought to determine the effects of age and vitamin E supplementation in the form of tocotrienol-rich fraction (TRF) on brain arginine metabolism. Male Wistar rats at ages of 3 and 21 months were supplemented with TRF orally for 3 months prior to the dissection of tissue from five brain regions. The tissue concentrations of L-arginine and its nine downstream metabolites were quantified using high-performance liquid chromatography and liquid chromatography tandem mass spectrometry. We found age-related alterations in L-arginine metabolites in the chemical- and region-specific manners. Moreover, TRF supplementation reversed age-associated changes in arginine metabolites in the entorhinal cortex and cerebellum. Multiple regression analysis revealed a number of significant neurochemical-behavioral correlations, indicating the beneficial effects of TRF supplementation on memory and motor function.

## 1. Introduction

Declined cognitive and motor functions occur during aging even in the absence of neurodegenerative diseases. While various mechanisms, such as cellular communication, oxidative stress, and inflammation, have been suggested to be involved in age-related cognitive and motor deficits [1], a growing body of evidence indicates the role of altered arginine metabolism in the aging and neurodegenerative processes [2–4].

L-Arginine is a semiessential amino acid that can be metabolized by nitric oxide synthase (NOS) to produce nitric oxide (NO) and L-citrulline, by arginase to form L-ornithine and urea, and by arginine decarboxylase (ADC) to generate agmatine and carbon dioxide [5]. NO plays an important role in maintaining physiological function of the nervous system [6]. However, it can be neurotoxic when present in excess due to its free radical property [7]. L-Ornithine is the main precursor of the polyamines putrescine, spermidine, and spermine [5], which are required for optimal cell growth and function [8], including neurogenesis [9]. L-Ornithine can also be converted to L-glutamyl-c-semialdehyde that is further metabolized to glutamate by P5C dehydrogenase [5]. Glutamate can also be synthesized by glutaminase using glutamine as a precursor [10] and can be converted to gamma-aminobutyric acid (GABA) by glutamate decarboxylase (GAD) [5]. Agmatine, decarboxylated arginine, is a novel putative neurotransmitter and directly participates in learning and memory processing [11–13]. It also plays an important role in regulating the production of NO and polyamines [14].

Given the physiological roles of L-arginine metabolites, a considerable number of studies have investigated how brain arginine metabolism is affected by aging. Earlier research has demonstrated age-related changes in the L-arginine metabolic profiles in the brain, particularly in the brain regions involved in learning and memory, in a region-specific manner, and the associations of neurochemical changes with animals' behavioral performance in various learning and memory tasks [2, 3, 15, 16]. It has been well documented that NO reacts with O_2_^−^ and H_2_O_2_ to form ONOO^−^, and the accumulation of these free radicals may lead to neuronal death and learning and memory impairments in the aged. To this end, pharmacological manipulations of brain L-arginine metabolism during aging may be a potential preventive or treatment strategy for age-related cognitive decline.

Neurodegeneration and cognitive decline can be modulated by dietary components rich in antioxidants such as vegetables and fruits and ascorbic acid [17–20]. Research has shown that supplementation with vitamin E is able to preserve cognitive function and general well-being in the elderly [21–23]. This is in accordance with the report that low plasma tocopherol and tocotrienol levels are associated with increased risks of mild cognitive impairment (MCI) and Alzheimer's disease (AD) [24]. Further investigation from our laboratory [25] provides additional evidence which showed 3-month daily supplementation of TRF able to reverse behavioral impairment in aged rats. In the study of Mangialasche et al. [26], elevated serum tocopherol and tocotrienol levels appear to be associated with reduced risk of cognitive impairment in older adults. Moreover, cell culture studies have demonstrated the neuroprotective effects of vitamin E, with tocotrienol being more potent than tocopherol [27–29]. The mechanisms of action of tocotrienol includes but not limited to free radical scavenging and modulation of arachidonic acid metabolism [28, 30–32], kainic acid metabolism [33], homocysteic acid metabolism [34], and mitochondrial metabolism [35].

The present study was therefore designed to further investigate how brain arginine metabolism was affected by age and to elucidate how the long-term supplementation of TRF affected brain arginine metabolism in both young and aged rats. The findings of this study demonstrated altered brain arginine metabolism in the aged rats in the chemical- and region-specific manners and the neuroprotective effect of vitamin E in the form of TRF via the modulation of brain L-arginine metabolism.

## 2. Materials and Methods

### 2.1. Animals

Thirty-six male Wistar rats were fed with standard rat chow (Gold Coin, Malaysia) and tap water ad libitum. Animals were housed one animal per cage at room temperature under a 12 h dark : light cycle. This study was approved by the Universiti Kebangsaan Malaysia Animal Ethics Committee (protocol nos. UKMAECFP/BIOK/2008/MUSALMAH/13-FEB/215-FEB-2008-OCT-2010).

### 2.2. Supplementation

Gold-Tri E70 (TRF) (Golden Hope Bioganic, Malaysia) consisted of approximately 149.2 mg/g *α*-tocopherol, 164.7 mg/g *α*-tocotrienol, 48.8 mg/g *β*-tocotrienol, 213.2 mg/g *γ*-tocotrienol, and 171 mg/g *δ*-tocotrienol. Olive oil was bought commercially (Basso, Italy). Rats were randomly divided into two groups: young and old (3 and 21 months, resp.). Rats in both groups were given either TRF (200 mg/kg body weight, oral gavage) or control (equal volume of olive oil, oral gavage) (*n* = 9 per group) daily, for 3 months. At the end of the treatment period, the rat's behavior was tested by the open-field test and Morris water maze task (see Taridi et al. [25]).

Evaluation on the locomotor activity, exploration, and anxiety of the rat in the open-field test was conducted by placing the rat into an open-field chamber (60 × 60 × 20 cm^3^). The floor of the open-field chamber was divided into 36 equal-sized square-shape cells (10 × 10 cm^2^). The rat's behavior was recorded within the 5 min exploration time. Then, the number of fecal boli, the number and duration of wall-supported rearing and grooming, the number and percentage of grid squares traversed, and the number of central squares crossed were analyzed.

To investigate spatial learning and memory, the Morris water maze (MWM) task was carried out in a black circular galvanized pool (140 cm diameter), filled with 30 cm water depth. The pool was divided into four equal quadrants, and a platform (13 × 13 cm) was placed in a target quadrant (the center of one of the quadrants), 2 cm below the water surface. This test was conducted within 10 consecutive days: place navigation (days 1 to 6), probe test (day 7), cued navigation (day 8), and working memory task (days 9 and 10). During the place navigation test, the rat was trained to find the fixed hidden platform. The parameters measured in this place navigation test were escape latency, swim path, and swim speed. On the next day after the completion of the place navigation test, the platform was removed and the rat was tested to find the platform location. This test was repeated twice for each rat. The percentage of time the rat spent in the target quadrant and the number of platform crossings in probe 1 (the first time the rat was tested without a platform) and probe 2 (the second time the rat was tested without a platform) were measured. Meanwhile, during the cued navigation test, the platform was shifted to another quadrant and raised 2 cm above the water surface. In order to make the platform more visible, the edge of the platform was marked by a masking tape. Then, the rat was allowed to find the visible platform within 60 s. Cued navigation is a control test to ensure that the rat used in this study has an intact vision. Escape latency was measured in this test.

On the next two days of Morris water maze, the working memory test was carried out. In this test, the rat was given two trials per day (each trial consisted of a sample phase and a test phase), with the hidden platform location changed between each trial. The sample phase involved in each trial the first time that the rat was placed in the pool. The rats were rested for 1 min and 1 hour before they were retested in the test phase. The parameters measured in this test were the path length taken by the rat to reach the platform on day 1 and day 2 (sample phase) and the path length during 1 min and 1 h delay time (test phase) (see Taridi et al. [25]).

### 2.3. Brain Tissue Collection and Preparation

Upon the completion of the behavioral test, animals were killed by decapitation without anesthesia. The brains were rapidly removed, wrapped in tin foil, immediately frozen on dry ice, and stored at −80°C. Before an assay, each frozen brain was sectioned at about 200 *μ*m in the coronal plane on dry ice. The cerebellum (CE), pons and medulla (brain stem (BS)), and striatum (only the tissue anterior to the anterior commissure) were collected. The entorhinal cortex (EC) and postrhinal cortex (POR) were then dissected out based on the previous studies [36–38] (see Figure 1 of Liu et al. [39]). Brain tissues were weighed, homogenized in 10% ice-cold perchloric acid (~50 mg wet weight per ml), and centrifuged at 10,000 rpm for 10 minutes at 4°C to precipitate protein [40]. The supernatants (perchloric acid extracts) were then frozen immediately and stored at −80°C until analysis using high-performance liquid chromatography (HPLC) and liquid chromatography tandem mass spectrometry (LC/MS/MS).

### 2.4. Standard and Reagent Preparation

The standard and reagent preparation for amino acid and polyamine analysis was done according to the method described previously [3, 40]. Briefly, aqueous stock solutions of amino acids and polyamines were prepared in double-distilled water with an initial concentration of 10 mM, except for glutamate (50 mM) and glutamine (200 mM). These stock solutions were then diluted with water. High-purity standards were used (Sigma, USA) and all other chemicals were of analytical grade. For amino acid analysis, KHCO_3_-KOH solution (pH 9.8), methanol, and dansyl chloride were added to the standards and samples. Dansyl chloride was prepared just before derivatization by dissolving it in acetonitrile. The mobile phase was 30 : 70 methanol/water (*v*/*v*) containing triethylamine and tetrabutylammonium hydroxide at pH 2.2. Saturated sodium carbonate, 1,7-diaminoheptane (internal standard), and dansyl chloride dissolved in acetone were added to the standards and samples for polyamine analysis. The mobile phase used for spermidine and spermine analysis was 70 : 30 acetonitrile/water and for agmatine and putrescine was 80 : 20 acetonitrile/water containing 0.1% formic acid. Samples from all groups were assayed at the same time for all brain regions and between all groups.

### 2.5. Amino Acid Analysis

L-Arginine, L-ornithine, glutamine, L-citrulline, glutamate, and GABA in each brain region were determined using HPLC (Shimadzu, Japan), as described previously [3]. The samples were alkalized with KHCO_3_ solution (pH 9.8), mixed with methanol, derivatized with dansyl chloride in the dark, and incubated at 80°C for 20 minutes, and 10 *μ*l acetic acid was added to stop the reaction. The mixture was then centrifuged at 10,000 rpm for 10 minutes, and 40 *μ*l of the resultant supernatant was injected onto the HPLC system. The HPLC system consisted of a programmed solvent delivery system at a flow rate of 1.0 ml/min and a UV detector set at a wavelength of 218 nm. The column used was reversed-phase C_18_ (5 *μ*m, 150 mm × 4.6 mm) (Phenomenex, USA). L-Arginine, L-ornithine, L-citrulline, glutamine, glutamate, and GABA in the brain samples were identified by comparing the retention time of the sample with the known standards. Amino acid concentrations were calculated with reference to the peak area of external standards, and values were expressed in *μ*g/g wet tissue.

### 2.6. Polyamine Analysis

Agmatine and putrescine concentrations were measured by a highly sensitive LC/MS/MS method according to the method described previously [40]. The internal standard was added to the samples, alkalized with saturated sodium carbonate, derivatized with dansyl chloride in the dark, and then incubated at 70°C for 30 minutes. Agmatine, putrescine, and the internal standard were extracted with toluene and centrifuged at 10,000 rpm for 5 minutes. The toluene phase was evaporated to dryness, reconstituted with 50% acetonitrile, and injected onto the LC/MS/MS system. The column used was reversed-phase C_18_ (5 *μ*m, 150 mm × 2.0 mm) (Phenomenex, USA). The mobile phase was 80% acetonitrile to 20% water containing 0.1% formic acid and was run at a flow rate of 0.2 ml/min. The retention time of agmatine, putrescine, and the internal standard was 1.7, 4.0, and 4.8 min, respectively, with 15 minutes of the total run time. Detection by MS/MS used an electrospray interface (ESI) in a positive ion mode. The standard curves for putrescine were linear up to 1000 ng/ml (*r*^2^ > 0.99). The intra- and interassay coefficients of variance were <15%. Agmatine and putrescine concentrations in the tissues were calculated with reference to the peak area of external standards, and values were expressed as *μ*g/g wet tissue.

Determination of spermidine and spermine was carried out according to Liu et al. [40]. Briefly, the internal standard (1,7-diaminoheptane) was added to 50 *μ*l of samples. Samples were then alkalized with saturated sodium carbonate, derivatized with dansyl chloride in the dark, and incubated at 70°C for 30 minutes. Spermidine, spermine, and the internal standard were extracted with toluene and centrifuged at 10,000 rpm for 5 minutes. The toluene phase was then evaporated to dryness, reconstituted with 50% acetonitrile, and injected onto the HPLC system. The HPLC system had a programmed solvent delivery system at a flow rate of 1.5 ml/min, an autosampler, a reversed-phase C_18_ column, and a fluorescence detector set at excitation wavelength of 252 nm and emission wavelength of 515 nm. Identification of spermidine and spermine was by comparing the retention times of samples with known standards. The precision of the intra- and interassay coefficients of variance was <15%. Spermidine and spermine concentrations in the brain tissue were calculated with reference to the peak area of external standards, and values were expressed as *μ*g/g wet tissue.

### 2.7. Statistical Analysis

Data was analyzed using two-way ANOVA, followed by the post hoc Bonferroni test to detect significant differences between groups by using GraphPad Prism 5 (GraphPad Software, USA). Data are presented as mean ± standard error mean (SEM). The significance level was set at 0.05 for all comparisons. Behavioral data (Morris water maze and open field) obtained previously [25] was analyzed for correlation with amino acids and polyamines in the different brain regions by multiple regression analysis. The significance value was set at *p* < 0.025.

## 3. Results

### 3.1. Effects of Age and TRF on Amino Acid Levels

**Figure 1:**
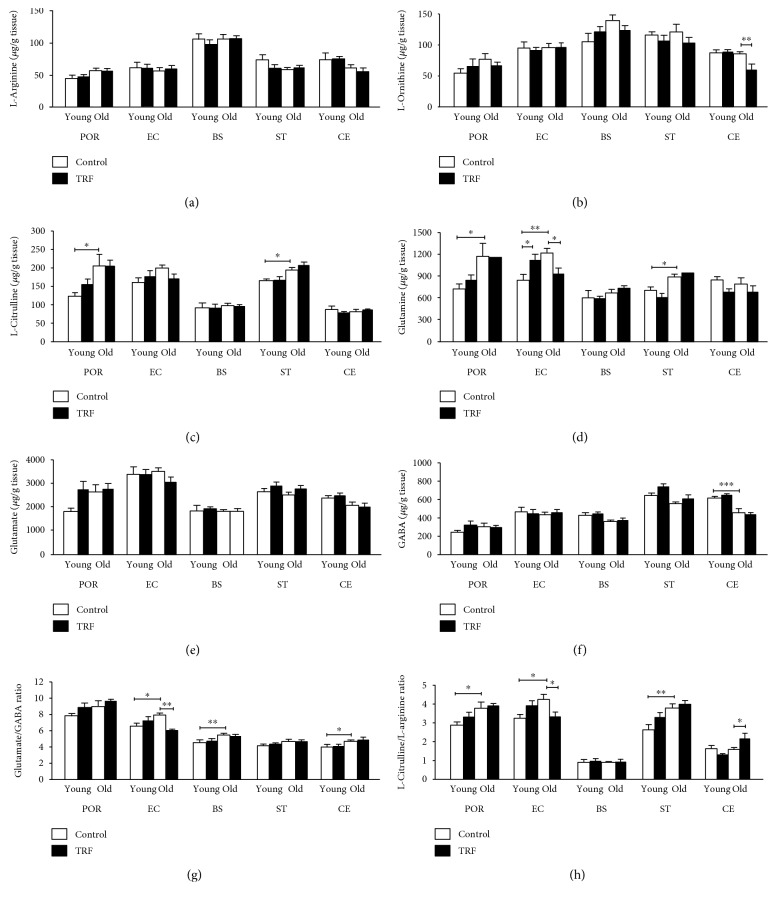
The levels of (a) L-arginine, (b) L-ornithine, (c) L-citrulline, (d) glutamine, (e) glutamate, (f) GABA, (g) glutamate/GABA ratio, and (h) L-citrulline/L-arginine ratio in the postrhinal cortex (POR), entorhinal cortex (EC), brain stem (BS), striatum (ST), and cerebellum (CE) in young and old rats. Data is presented as mean ± SEM. The asterisks indicate a significant difference between groups: ^∗^*p* < 0.05, ^∗∗^*p* < 0.01, and ^∗∗∗^*p* < 0.001.

The current findings showed that the changes in the amino acid level were primarily affected by age. All amino acid levels were altered with age except L-arginine and glutamate. In addition, TRF supplementation modulates the level of L-ornithine, glutamine, glutamate/GABA ratio, and L-citrulline/L-arginine ratio in several brain regions. L-Arginine in the different brain regions across all groups are presented in Figure 1(a). There was no significant effect of age or TRF supplementation observed.  Figure 1(b) represents the L-ornithine in different areas of the brain. There was no significant effect of age; however, TRF supplementation significantly decreased the L-ornithine level in the cerebellum of old rats compared to the control (unsupplemented) (*p* < 0.01). L-Citrulline was significantly higher in the postrhinal cortex (POR) and striatum (ST) (*p* < 0.05) of old versus young rats (Figure 1(c)). L-Citrulline was unchanged with TRF supplementation in all groups. Age significantly increased glutamine in the postrhinal cortex (*p* < 0.05), entorhinal cortex (*p* < 0.01), and striatum (*p* < 0.05) (Figure 1(d)). TRF supplementation resulted in an increase in glutamine in the entorhinal cortex of young rats (*p* < 0.05) but was decreased in the old rats (*p* < 0.05). There was no significant effect of age nor TRF supplementation on glutamate (Figure 1(e)).  Figure 1(f) shows that there was a significant effect of age on GABA in the cerebellum. The level of GABA was significantly lower in the old rats compared to the young rats (*p* < 0.001).

The glutamate/GABA was significantly increased in the entorhinal cortex (*p* < 0.05), brain stem (*p* < 0.01), and cerebellum (*p* < 0.05) of the old rats compared to the young rats (Figure 1(g)). TRF supplementation decreased glutamate/GABA significantly in the entorhinal cortex of the old group (*p* < 0.01). Effects of age were also observed with L-citrulline/L-arginine in the postrhinal cortex (*p* < 0.05), entorhinal cortex (*p* < 0.05), and striatum (*p* < 0.01) (Figure 1(h)). TRF supplementation significantly decreased the L-citrulline/L-arginine ratio in the entorhinal cortex (*p* < 0.05) but was increased in the cerebellum of the old rats (*p* < 0.05).

### 3.2. Effects of Age and TRF on Polyamines

**Figure 2:**
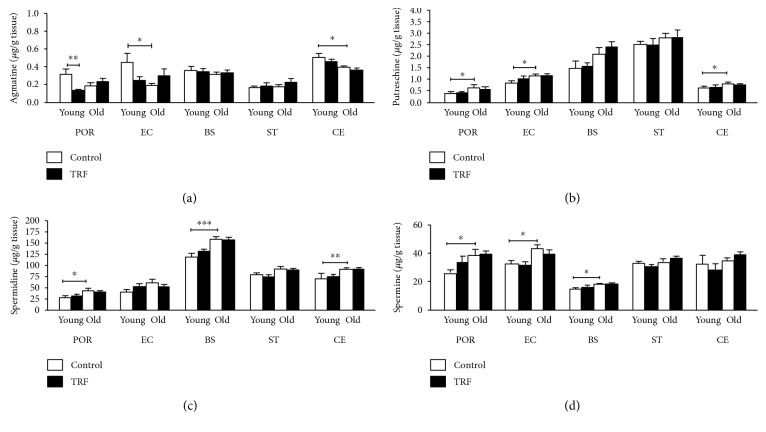
The levels of (a) agmatine, (b) putrescine, (c) spermidine, and (d) spermine in the postrhinal cortex (POR), entorhinal cortex (EC), brain stem (BS), striatum (ST), and cerebellum (CE) in young and old rats. Data is presented as mean ± SEM. The asterisks indicate a significant difference between groups: ^∗^*p* < 0.05, ^∗∗^*p* < 0.01, and ^∗∗∗^*p* < 0.001.

**Figure 3:**
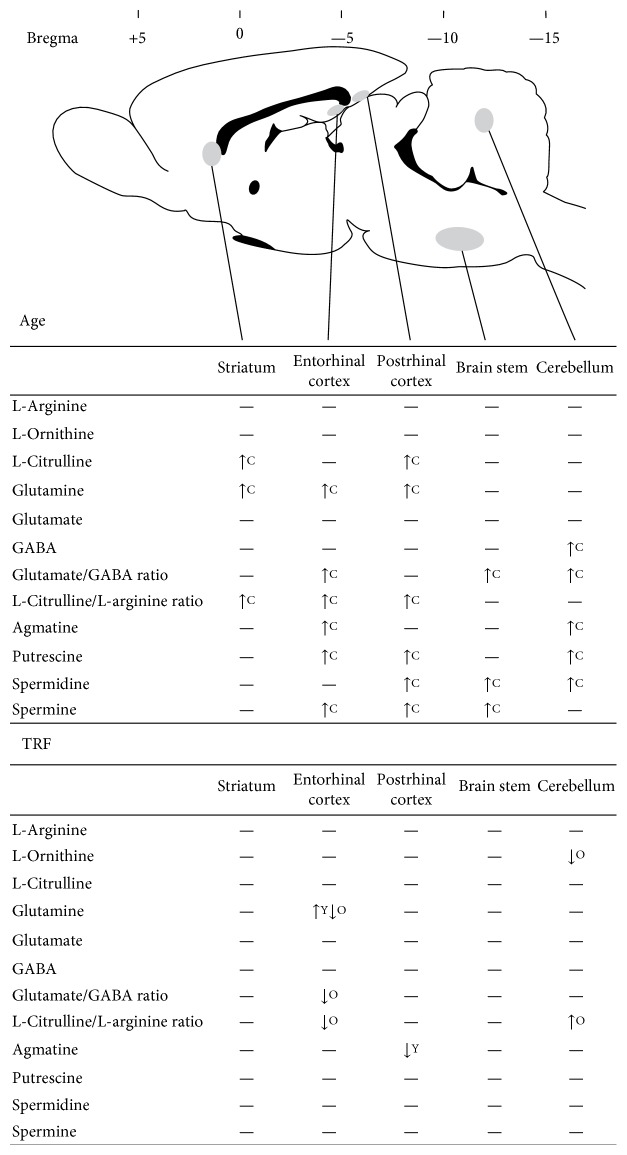
Summary of the effect of age and TRF on amino acid (L-arginine, L-ornithine, L-citrulline, glutamine, glutamate, GABA, glutamate/GABA ratio, and L-citrulline/L-arginine ratio) and polyamine (agmatine, putrescine, spermidine, and spermine) levels in the striatum, postrhinal cortex, entorhinal cortex, brain stem, and cerebellum. Decreased levels are shown by downward arrows and increased levels by upward arrows. No changes are indicated by a horizontal dash. C represents the effect of age on control groups; Y represents the effect of TRF on young groups; O represents the effect of TRF on old groups. Adapted from the rat brain atlas [67].

Alterations in the polyamine levels were mainly influenced by age. Age affects all polyamines in various regions, while TRF supplementation only caused an alteration in the agmatine level only in the postrhinal cortex.  Figure 2(a) shows that agmatine was significantly lower in the entorhinal cortex (*p* < 0.05) and cerebellum (*p* < 0.05) of old rats compared to young rats. TRF supplementation resulted in decreased agmatine in the postrhinal cortex (*p* < 0.01) of young rats. Putrescine was significantly increased in the postrhinal cortex (*p* < 0.05), entorhinal cortex (*p* < 0.05), and cerebellum (*p* < 0.05) of old rats compared to young rats (Figure 2(b)). Spermidine was increased in the old group compared to the young group in the postrhinal cortex (*p* < 0.05), brain stem (*p* < 0.001), and cerebellum (*p* < 0.01) (Figure 2(c)). Spermine was significantly increased in the postrhinal cortex (*p* < 0.05), entorhinal cortex (*p* < 0.05), and brain stem (*p* < 0.05) (Figure 2(d)).  Figure 3 shows the summary of the effects of age and TRF supplementation on amino acids and polyamines in the different parts of the brain.

### 3.3. Correlations between Behavior, Amino Acids, and Polyamines

Based on the previous findings by Taridi et al. [25], TRF supplementation to the aged rats markedly improved the animals' behavioral performance in the open-field and Morris water maze tests, as shown by the increased exploratory activity and improved spatial learning and memory, respectively. Therefore, the current study further analyzed the behavioral performance data in relation to the neurochemical level in the rats' brain, to further explore the effects of age on particular behavioral parameters as well as to elucidate the potential neuroprotective effect of TRF.

**Figure 4:**
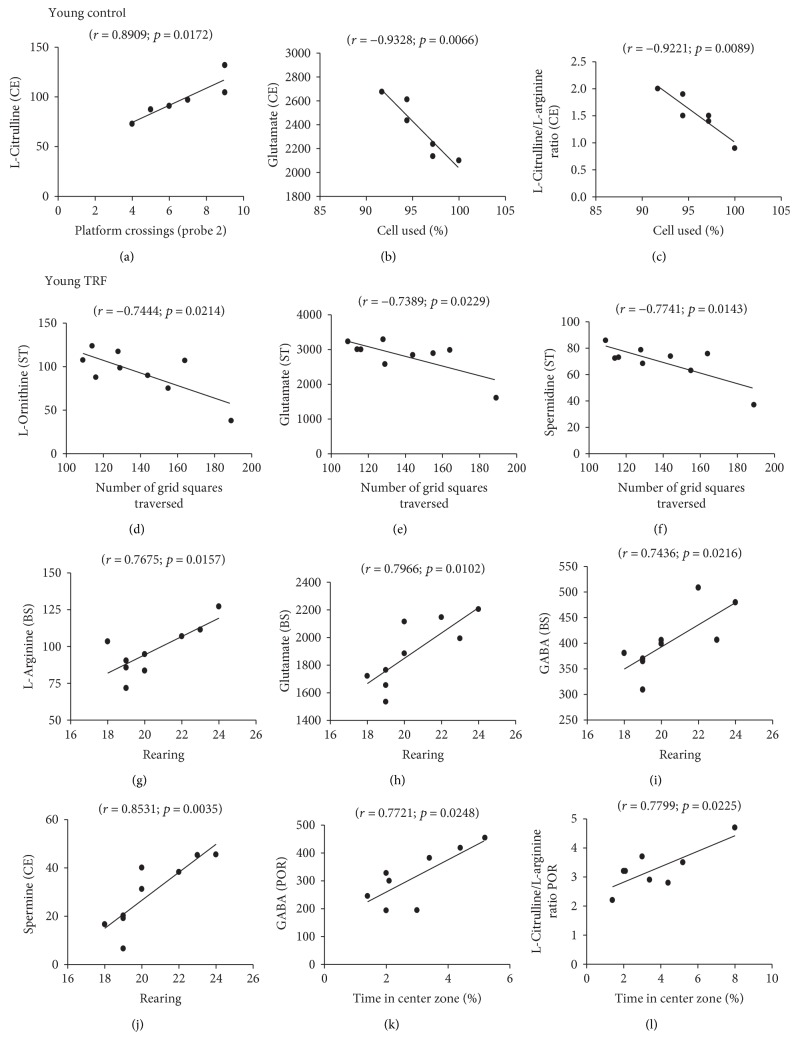
Scattergrams of the significant correlations between behavioral measures and neurochemical variables in the postrhinal cortex, entorhinal cortex, brain stem, striatum, and cerebellum in young rats.

**Figure 5:**
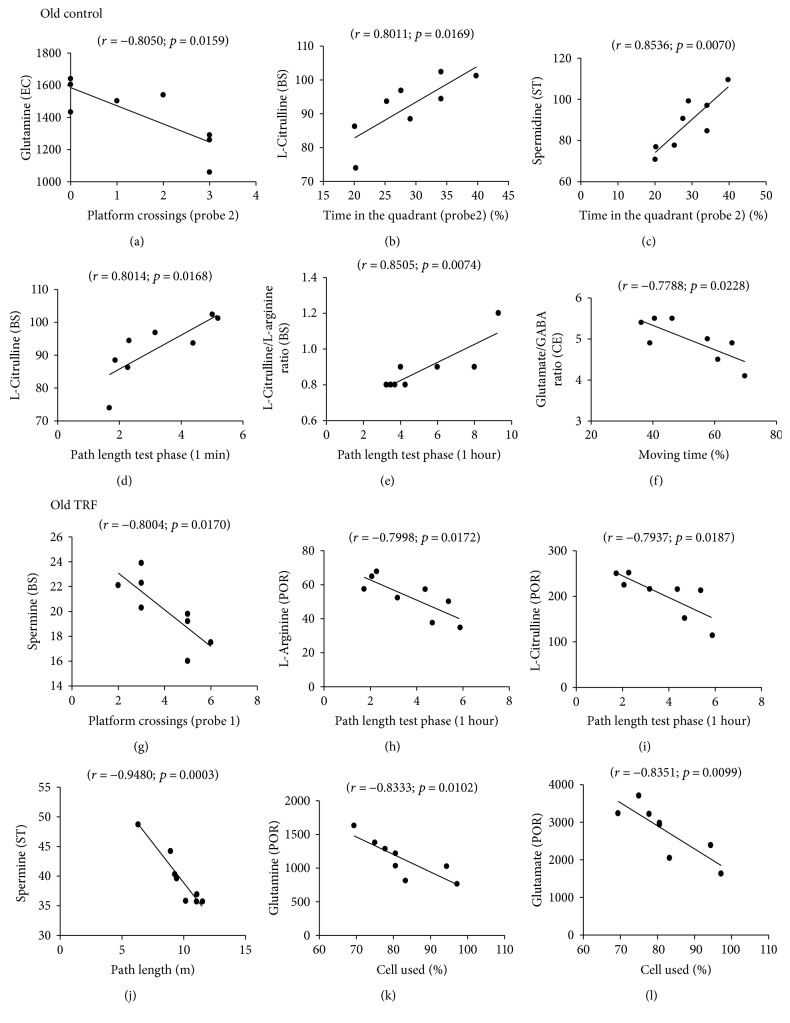
Scattergrams of the significant correlations between behavioral measures and neurochemical variables in the postrhinal cortex, entorhinal cortex, brain stem, striatum, and cerebellum in old rats.

The relationships between the levels of amino acids and polyamines and the animals' behavioral performance in the open-field and Morris water maze tests were analyzed using multiple regression analysis. Only those with significant correlations in each of the test group are shown in Figures 4 and 5. In the young control group, cerebellum L-citrulline was positively correlated with the number of platform crossings during probe 2 (*r* = 0.8909, *p* = 0.0172; Figure 4(a)) indicating better memory. However, cerebellum glutamate (*r* = −0.9328, *p* = 0.0066; Figure 4(b)) and L-citrulline/L-arginine (*r* = −0.9221, *p* = 0.0089; Figure 4(c)) were negatively correlated with the percentage of cells used during the open field indicating decreased locomotor activity.

In the TRF-supplemented young group, negative correlations were observed between striatum L-ornithine (*r* = −0.7444, *p* = 0.0214; Figure 4(d)), glutamate (*r* = −0.7389, *p* = 0.0229; Figure 4(e)), and spermidine (*r* = −0.7741, *p* = 0.0143; Figure 4(f)) and the number of grid squares traversed by the rats during the open field. However, positive correlations were observed between brain stem L-arginine (*r* = 0.7675, *p* = 0.0157; Figure 4(g)), glutamate (*r* = 0.7966, *p* = 0.0102; Figure 4(h)), and GABA (*r* = 0.7436, *p* = 0.0216; Figure 4(i)) and cerebellum spermine (*r* = 0.8531, *p* = 0.0035, Figure 4(j)) and the number of rearing during the open field. Postrhinal cortex GABA (*r* = 0.7721, *p* = 0.0248; Figure 4(k)) and L-citrulline/L-arginine (*r* = 0.7799, *p* = 0.0225; Figure 4(l)) were positively correlated with the percentage of time the rats spent in the center zone during the open-field test.

In the old control group, entorhinal cortex glutamine (*r* = −0.8050, *p* = 0.0159; Figure 5(a)) was negatively correlated with the number of platform crossings during the water maze probe test. Brain stem L-citrulline (*r* = 0.8011, *p* = 0.0169; Figure 5(b)) and striatum spermidine (*r* = 0.8536; *p* = 0.0070; Figure 5(c)) were positively correlated with the percentage of time the rats spent in the target quadrant during the water maze probe test. Positive correlations were also observed between brain stem L-citrulline (*r* = 0.8014, *p* = 0.0168; Figure 5(d)) and L-citrulline/L-arginine (*r* = 0.8505, *p* = 0.0074; Figure 5(e)) and the mean path length used by the rats to reach the hidden platform during the working memory test. Cerebellum glutamate/GABA was negatively correlated with the percentage of time the rats moved during the open-field test (*r* = −0.7788, *p* = 0.0228; Figure 5(f)).

In the TRF-supplemented old group, negative correlations were observed between brain stem spermine (*r* = −0.8004, *p* = 0.0170; Figure 5(g)) and L-arginine (*r* = −0.7998, *p* = 0.0172; Figure 5(h)) and postrhinal cortex L-citrulline (*r* = −0.7937, *p* = 0.0187; Figure 5(i)) and the number of platform crossings during the probe test and the mean of path length used during the working memory test phase for 1 hour, respectively. Striatum spermine (*r* = −0.9480, *p* = 0.0003; Figure 5(j)) and glutamine (*r* = −0.8333, *p* = 0.0102; Figure 5(k)) and postrhinal cortex glutamate (*r* = −0.8351, *p* = 0.0099; Figure 5(l)) were negatively correlated with the mean of path length and the percentage of cell used, respectively. These results showed that changes in the amino acid and polyamine levels in the specific regions of the brain were associated with the rats' cognitive and locomotor functions.

## 4. Discussion

The present study investigated the effects of age and TRF supplementation on L-arginine and its metabolites in the postrhinal cortex, entorhinal cortex, brain stem, striatum, and cerebellum of young and old rats. The findings concurred with those of the previous reports that age-related changes in L-arginine metabolism in the brain are region specific [2, 3, 16].  Figure 3 shows that the postrhinal cortex, entorhinal cortex, and cerebellum were the regions most affected by age. The postrhinal cortex and entorhinal cortex play critical roles in memory processing. The postrhinal cortex is the area involved in spatial memory [41], while the entorhinal cortex receives spatial and nonspatial information from the postrhinal and perirhinal cortex, respectively [42]. The cerebellum is the area involved in motor control [43] as well as in emotion and cognition [44, 45]. Thus, alterations of the neurochemicals in these areas may affect memory processing, leading to cognitive impairment with age.

Age-related alterations in glutamine, L-citrulline, GABA, glutamate/GABA, and L-citrulline/L-arginine, as well as in levels of polyamines agmatine, putrescine, spermidine, and spermine were observed in the present study (Figure 3). Glutamine and L-citrulline increased with age in the postrhinal cortex and striatum while GABA decreased with age in the cerebellum. Glutamate/GABA increased with age in the entorhinal cortex, brain stem, and cerebellum. The L-citrulline/L-arginine ratio was increased in the striatum, postrhinal cortex, and entorhinal cortex.

Glutamine is the most abundant free amino acid in the human blood and regulates a variety of target genes involved in cell proliferation, differentiation, and survival [46]. Glutamine is produced by glutamine synthetase (GS), which catalyzes the formation of glutamine from glutamate and ammonia [47]. Thus, increased glutamine in aged rats may be a consequence of increased GS activity. Glutamine serves as a precursor for glutamate and hence is involved in energy production [48]. Changes in glutamine-glutamate cycle flux therefore could affect brain mitochondrial metabolism during aging [49].

In the present study, an age-related increase in L-citrulline was observed in the postrhinal cortex (Figure 1(c)) and striatum (Figure 1(c)), even though its precursors, L-arginine and L-ornithine, were largely unaffected. L-Citrulline is formed from L-ornithine by OTC as well as from L-arginine by NOS. Thus, alterations in L-citrulline level may reflect changes in the activities of NOS and/or OTC with age. Increased NOS activity with age had been reported in the postrhinal and entorhinal cortex [2, 3]. Conversion of L-citrulline to L-arginine by argininosuccinate synthase (ASS) and argininosuccinate lyase (ASL) [5] may explain why L-arginine is not affected by age. Increased L-citrulline without any changes in the L-arginine results in the increase in the L-citrulline/L-arginine observed. Increased glutamate/GABA in the entorhinal cortex, brain stem, and cerebellum (Figure 1(g)) may indicate changes in the glutamatergic and GABAergic systems which have been suggested to alter cognitive functions [50–52].

Polyamines are ubiquitous small molecules that play essential roles in learning and memory [53]. The effect of age on polyamines was also noted to be region specific (Figure 3). These changes may reflect the alterations of the activities of the enzymes involved in their synthesis. L-Arginine and L-ornithine are considered the main precursors of polyamines. Putrescine is converted to spermidine by spermidine synthase (SRM), which is then converted to spermine by spermine synthase (SMS). In this study, only agmatine was observed to decrease with age. Possible explanations may include the roles of agmatine as competitive inhibitors for NOS [54] and ODC [5, 55].

The present findings agreed with those of the earlier reports on the effect of age on amino acids [16, 56] and polyamines [16, 40]. However, there were some differences observed: glutamate and GABA in the brain stem [57] and L-arginine, L-ornithine, L-citrulline, glutamate, GABA, putrescine, spermidine, spermine, and agmatine in the various parts of the brain [2, 3, 16]. Discrepancies in the findings on L-arginine and its metabolites in young and aged brains have also been reported previously. For example, decreased glutamate was reported in the rat entorhinal cortex [2, 3], the whole cerebral cortex [58–60], and the frontal region [61–63] while others reported increased levels [64]. Some authors have attributed these differences to differences in the animals' experience (with and without behavioral testing and treatment) [2, 3, 16].

Accumulation of polyamines can be toxic to the cells. Normally, cell protects itself against toxic accumulation of polyamines by synthesizing an antizyme, a regulatory protein that can inhibit ODC activity [65]. However, protein oxidation during aging could diminish the synthesis of an antizyme, leading to the accumulation of these polyamines to toxic level. A free radical scavenger such as vitamin E is suggested to prevent the degradation of an antizyme by quenching free radicals [66]. In the present study, TRF supplementation was observed to reduce some amino acids primarily in the entorhinal cortex and the cerebellum (Figure 3). However, TRF supplementation did not result in any changes in polyamines except for agmatine in the young rats suggesting that the neuroprotective effects of TRF could be mediated by the modulation of L-arginine metabolism rather than its antioxidant activity.

The present data shows that TRF supplementation in old rats reversed the age effect on glutamine, as well as on glutamate/GABA and L-citrulline/L-arginine in the entorhinal cortex. Glutamine is a precursor for an excitatory neurotransmitter, glutamate, while the entorhinal cortex plays a vital role in memory processing. Thus, the alteration of glutamine in the entorhinal cortex with TRF supplementation may possibly represent the involvement of TRF in modulating learning and memory. Apart from the entorhinal cortex, TRF also modulated L-ornithine and hence L-citrulline/L-arginine in the cerebellum. L-Ornithine is a precursor for putrescine and L-citrulline, catalyzed by ODC and OTC, respectively. Conversion of L-ornithine to L-glutamyl-c-semialdehyde can be further metabolized by P5C dehydrogenase for glutamate synthesis [5]. TRF modulation on L-ornithine in the cerebellum may affect cognition and locomotor activity in old rats. On the other hand, modulation of TRF on glutamate/GABA and L-citrulline/L-arginine suggested the involvement of TRF in maintaining brain glutamate, GABA, L-citrulline, and L-arginine balance.

Amino acids and polyamines were further correlated with behavioral variables observed with the same rats under the same experimental procedure, and the findings were published previously [25]. The behavioral studies were carried out using the open-field test and the Morris water maze test. The open-field tests were for the animals' locomotor activity while the Morris water maze tests for memory retention. Multiple regression analysis revealed a number of significant correlations between amino acid and polyamine levels and the rats' behavior in each group. In the young control group, higher L-citrulline in the cerebellum was correlated with better performance in the water maze (better memory). Lower glutamate and L-citrulline/L-arginine in the cerebellum were correlated with higher locomotor activity in the open field. It is interesting to note that only changes in the cerebellum amino acids influenced the cognitive behavior in the young group.

The data from this study indicate that with age, more neurochemicals from wider brain areas influenced behavior. In the old control group, poor memory was correlated with higher entorhinal cortex glutamate and glutamine; higher brain stem L-citrulline, glutamine, and L-citrulline/L-arginine; and lower brain stem L-citrulline and striatum spermidine. Higher cerebellum glutamate/GABA was correlated with the lower percentage of movement (lower locomotor activity) in the open-field test.

TRF supplementation in the young triggers the involvement of more neurochemicals from different areas of the brain to influence behavior, particularly the locomotor activity. Lower striatum L-ornithine, glutamate, and spermidine and higher brain stem L-arginine, glutamate, and GABA; cerebellum spermine; and postrhinal cortex GABA and L-citrulline/L-arginine were correlated with better performance in the open-field test (higher locomotor activity). However, TRF was able to influence both memory and locomotor activity in the old group. Lower brain stem spermine and higher postrhinal cortex L-arginine and L-citrulline were correlated with better performance in the water maze (better memory) while lower striatum spermine and postrhinal cortex glutamine and glutamate were correlated with better performance in the open-field test (higher locomotor activity). Hence, the data shows that neurotransmitters affect behavior and cognition and that this can be influenced by age and TRF supplementation.

## 5. Conclusion

In conclusion, the present study shows that age-related changes in L-arginine and its metabolites are region specific in the brain. TRF supplementation was able to reverse some of the age-related alterations in amino acids of the brain regions involved in both memory processing and motor control.

## Abbreviations

TRF: Tocotrienol-rich fraction
NOS: Nitric oxide synthase
NOS: Nitric oxide
ADC: Arginine decarboxylase
GABA: Gamma-aminobutyric acid
GAD: Glutamate decarboxylase
MCI: Mild cognitive impairment
AD: Alzheimer's disease
HPLC: High-performance liquid chromatography
LC/MS/MS: Liquid chromatography tandem mass spectrometry
ESI: Electrospray interface
GS: Glutamine synthetase
ASS: Argininosuccinate synthase
ASL: Argininosuccinate lyase
SRM: Spermidine synthase
SMS: Spermine synthase
ODC: Ornithine decarboxylase
OTC: Ornithine transcarbamylase.

## References

[1] Yankner B. A., Lu T., Loerch P. (2008). The aging brain. *Annual Review of Pathology: Mechanisms of Disease*.

[2] Gupta N., Jing Y., Collie N. D., Zhang H., Liu P. (2012). Ageing alters behavioural function and brain arginine metabolism in male Sprague-Dawley rats. *Neuroscience*.

[3] Liu P., Jing Y., Zhang H. (2009). Age-related changes in arginine and its metabolites in memory-associated brain structures. *Neuroscience*.

[4] Liu P., Fleete M. S., Jing Y. (2014). Altered arginine metabolism in Alzheimer’s disease brains. *Neurobiology of Aging*.

[5] Wu G., Morris S. M. (1998). Arginine metabolism: nitric oxide and beyond. *Biochemical Journal*.

[6] Calabrese V., Boyd-Kimball D., Scapagnini G., Butterfield D. A. (2004). Nitric oxide and cellular stress response in brain aging and neurodegenerative disorders: the role of vitagenes. *In Vivo*.

[7] Wiesinger H. (2001). Arginine metabolism and the synthesis of nitric oxide in the nervous system. *Progress in Neurobiology*.

[8] Wallace H. M., Fraser A. V., Hughes A. (2003). A perspective of polyamine metabolism. *Biochemical Journal*.

[9] Malaterre J., Strambi C., Aouane A., Strambi A., Rougon G., Cayre M. (2004). A novel role for polyamines in adult neurogenesis in rodent brain. *European Journal of Neuroscience*.

[10] Kaiser L. G., Schuff N., Cashdollar N., Weiner M. W. (2005). Age-related glutamate and glutamine concentration changes in normal human brain: ^1^H MR spectroscopy study at 4 T. *Neurobiology of Aging*.

[11] Leitch B., Shevtsova O., Reusch K., Bergin D. H., Liu P. (2011). Spatial learning-induced increase in agmatine levels at hippocampal CA1 synapses. *Synapse*.

[12] Liu P., Collie N. D., Chary S., Jing Y., Zhang H. (2008). Spatial learning results in elevated agmatine levels in the rat brain. *Hippocampus*.

[13] McKay B. E., Lado W. E., Martin L. J., Galic M. A., Fournier N. M. (2002). Learning and memory in agmatine-treated rats. *Pharmacology Biochemistry and Behavior*.

[14] Halaris A., Piletz J. (2007). Agmatine: metabolic pathway and spectrum of activity in brain. *CNS Drugs*.

[15] Cassel J. C., Schweizer T., Lazaris A. (2005). Cognitive deficits in aged rats correlate with levels of L-arginine, not with nNOS expression or 3,4-DAP-evoked transmitter release in the frontoparietal cortex. *European Neuropsychopharmacology*.

[16] Rushaidhi M., Jing Y., Kennard J. (2012). Aging affects L-arginine and its metabolites in memory-associated brain structures at the tissue and synaptoneurosome levels. *Neuroscience*.

[17] Harrison F. E., Bowman G. L., Polidori M. C. (2014). Ascorbic acid and the brain: rationale for the use against cognitive decline. *Nutrients*.

[18] Joseph J. A., Shukitt-Hale B., Denisova N. A. (1999). Reversals of age-related declines in neuronal signal transduction, cognitive, and motor behavioral deficits with blueberry, spinach, or strawberry dietary supplementation. *The Journal of Neuroscience*.

[19] Jovanoviv Z. (2014). Antioxidative Defense Mechanisms in the Aging Brain. *Archives of Biological Sciences*.

[20] Willis L. M., Shukitt-Hale B., Joseph J. A. (2009). Modulation of cognition and behavior in aged animals: role for antioxidant- and essential fatty acid-rich plant foods. *The American Journal of Clinical Nutrition*.

[21] Dysken M. W., Sano M., Asthana S. (2014). Effect of vitamin E and memantine on functional decline in Alzheimer disease: the team-AD VA cooperative randomized trial. *Journal of the American Medical Association*.

[22] Morris M. C., Evans D. A., Bienias J. L., Tangney C. C., Wilson R. S. (2002). Vitamin E and cognitive decline in older persons. *Archives of Neurology*.

[23] Morris M. C., Evans D. A., Tangney C. C. (2005). Relation of the tocopherol forms to incident Alzheimer disease and to cognitive change. *The American Journal of Clinical Nutrition*.

[24] Mangialasche F., Xu W., Kivipelto M. (2012). Tocopherols and tocotrienols plasma levels are associated with cognitive impairment. *Neurobiology of Aging*.

[25] Taridi N. M., Ab Rani N., Abdul Latiff A. A., Wan Ngah W. Z., Mazlan M. (2014). Tocotrienol rich fraction reverses age-related deficits in spatial learning and memory in aged rats. *Lipids*.

[26] Mangialasche F., Solomon A., Kareholt I. (2013). Serum levels of vitamin E forms and risk of cognitive impairment in a Finnish cohort of older adults. *Experimental Gerontology*.

[27] Mazlan M., Then S. M., Wan Ngah W. Z. (2006). Comparative effects of *α*-tocopherol and *γ*-tocotrienol against hydrogen peroxide induced apoptosis on primary-cultured astrocytes. *Journal of the Neurological Sciences*.

[28] Sen C. K., Khanna S., Roy S., Packer L. (2000). Molecular basis of vitamin E action: tocotrienol potently inhibits glutamate-induced pp60c-Src kinase activation and death of HT4 neuronal cells. *Journal of Biological Chemistry*.

[29] Then S. M., Wan Ngah W. Z., Mat Top G., Mazlan M. (2010). Comparison of the effects of *α*-tocopherol and *γ*-tocotrienol against oxidative stress in two different neuronal cultures. *Sains Malaysiana*.

[30] Khanna S., Roy S., Ryu H. (2003). Molecular basis of vitamin E action: tocotrienol modulates 12-lipoxygenase, a key mediator of glutamate-induced neurodegeneration. *Journal of Biological Chemistry*.

[31] Khanna S., Parinandi N. L., Kotha S. R. (2010). Nanomolar vitamin E *α*-tocotrienol inhibits glutamate-induced activation of phospholipase A2 and causes neuroprotection. *Journal of Neurochemistry*.

[32] Rink C., Khanna S., Sen C. K., Basu S., Wiklund L. (2011). Arachidonic acid metabolism and lipid peroxidation in stroke: alpha-tocotrienol as a unique therapeutic agent. *Studies on Experimental Models*.

[33] Jung N. Y., Lee K. H., Won R., Lee B. H. (2013). Neuroprotective effects of *α*-tocotrienol on kainic acid-induced neurotoxicity in organotypic hippocampal slice cultures. *International Journal of Molecular Sciences*.

[34] Khanna S., Roy S., Parinandi N. L., Maurer M., Sen C. K. (2006). Characterization of the potent neuroprotective properties of the natural vitamin E *α*-tocotrienol. *Journal of Neurochemistry*.

[35] Navarro A., Boveris A. (2010). Brain mitochondrial dysfunction in aging, neurodegeneration, and Parkinson’s disease. *Frontiers in Aging Neuroscience*.

[36] Burwell R. D., Witter M. P., Amaral D. J. (1995). Perirhinal and postrhinal cortices of the rats: a review of the neuroanatomical literature and comparison with findings from the monkey brain. *Hippocampus*.

[37] Burwell R. D. (2001). Borders and cytoarchitecture of the perirhinal and postrhinal cortices in the rat. *The Journal of Comparative Neurology*.

[38] Paxinos G., Watson C. (1998). *The Rat Brain in Stereotaxic Coordinates*.

[39] Liu P., Smith P. F., Appleton I., Darlington C. L., Bilkey D. K. (2003). Nitric oxide synthase and arginase in the rat hippocampus and the entorhinal, perirhinal, postrhinal, and temporal cortices: regional variations and age-related changes. *Hippocampus*.

[40] Liu P., Gupta N., Jing Y., Zhang H. (2008). Age-related changes in polyamines in memory-associated brain structures in rats. *Neuroscience*.

[41] Agster K. L., Burwell R. D. (2009). Cortical efferents of the perirhinal, postrhinal, and entorhinal cortices of the rat. *Hippocampus*.

[42] Squire L. R., Stark C. E., Clark R. E. (2004). The medial temporal lobe. *Annual Review of Neuroscience*.

[43] Augustin I., Korte S., Rickmann M. (2001). The cerebellum-specific Munc13 isoform Munc13-3 regulates cerebellar synaptic transmission and motor learning in mice. *The Journal of Neuroscience*.

[44] Schmahmann J. D., Caplan D. (2006). Cognition, emotion and the cerebellum. *Brain*.

[45] Schutter D. J., van Honk J. (2005). The cerebellum on the rise in human emotion. *Cerebellum*.

[46] Brasse-Lagnel C., Lavoinne A., Husson A. (2009). Control of mammalian gene expression by amino acids, especially glutamine. *FEBS Journal*.

[47] Albrecht J., Sonnewald U., Waagepetersen H. S., Schousboe A. (2007). Glutamine in the central nervous system: function and dysfunction. *Frontiers in Bioscience*.

[48] Kvamme E., Roberg B., Torgner I. A. (2000). Glutamine transport in brain mitochondria. *Neurochemistry International*.

[49] Boumezbeur F., Mason G. F., de Graaf R. A. (2009). Altered brain mitochondrial metabolism in healthy aging as assessed by in vivo magnetic resonance spectroscopy. *Journal of Cerebral Blood Flow and Metabolism*.

[50] Hardy J., Adolfsson R., Alafuzoff I. (1985). Transmitter deficits in Alzheimer’s disease. *Neurochemistry International*.

[51] Lowe S. L., Francis P. T., Procter A. W. (1988). Gamma-aminobutyric acid concentration in brain tissue at two stages of Alzheimer’s disease. *Brain*.

[52] Reinikainen K. J., Paljarvi L., Huuskonen M. (1988). A post-mortem study of noradrenergic, serotonergic and GABAergic neurons in Alzheimer’s disease. *Journal of the Neurological Sciences*.

[53] Williams K. (1997). Interactions of polyamines with ion channels. *Biochemical Journal*.

[54] Galea E., Regunathan S., Eliopoulos V., Feinstein D. L., Reis D. J. (1996). Inhibition of mammalian nitric oxide synthases by agmatine, an endogenous polyamine formed by decarboxylation of arginine. *Biochemical Journal*.

[55] Satriano J., Matsufuji S., Murakami Y. (1998). Agmatine suppresses proliferation by frameshift induction of antizyme and attenuation of cellular polyamine levels. *Journal of Biological Chemistry*.

[56] Liu P., Zhang H., Devaraj R., Ganesalingam G. S., Smith P. F. (2010). A multivariate analysis of the effects of aging on glutamate, GABA and arginine metabolites in the rat vestibular nucleus. *Hearing Research*.

[57] Jing Y., Fleete M. S., Collie N. D., Zhang H., Liu P. (2013). Regional variations and age-related changes in arginine metabolism in the rat brain stem and spinal cord. *Neuroscience*.

[58] Saransaari P., Oja S. S. (1995). Age-related changes in the uptake and release of glutamate and aspartate in the mouse brain. *Mechanisms of Ageing and Development*.

[59] Strolin-Benedetti M., Cini M., Fusi R., Marrari P., Dostert P. (1990). The effects of aging on MAO activity and amino acid levels in rat brain. *Journal of Neural Transmission*.

[60] Strolin-Benedetti M., Ruso A., Marrari P., Dostert P. (1991). Effects of aging on the content in sulfur-containing amino acids in rat brain. *Journal of Neural Transmission*.

[61] Dawson R., Wallace D. R., Meldrum M. J. (1989). Endogenous glutamate release from frontal cortex of adult and aged rats. *Neurobiology of Aging*.

[62] Fornieles F., Peinado J. M., Mora F. (1986). Endogenous levels of amino acid neurotransmitters in different regions of frontal and temporal cortex of the rat during the normal process of aging. *Neuroscience Letters*.

[63] Wallace D. R., Dawson R. (1990). Effect of age and monosodium-L-glutamate (MSG) treatment on neurotransmitter content in brain regions from male Fischer-344 rats. *Neurochemical Research*.

[64] Messripour M., Mesripour A. (2011). Effects of vitamin B6 on age associated changes of rat brain glutamate decarboxylase activity. *African Journal of Pharmacy and Pharmacology*.

[65] Hayashi S., Murakami Y. (1995). Rapid and regulated degradation of ornithine decarboxylase. *Biochemical Journal*.

[66] Yatin S. M., Yatin M., Aulick T., Ain K. B., Butterfield D. A. (1999). Alzheimer’s amyloid *β*-peptide associated free radicals increase rat embryonic neuronal polyamine uptake and ornithine decarboxylase activity: protective effect of vitamin E. *Neuroscience Letters*.

[67] Paxinos G., Watson C. (2014). *The Rat Brain in Stereotaxic Coordinates*.

